# Novel predictors of late recurrence after catheter ablation for atrial fibrillation: from biomarkers to artificial intelligence models

**DOI:** 10.3389/fcvm.2026.1831993

**Published:** 2026-09-03

**Authors:** Chiyu Chen

**Affiliations:** 1 Zhejiang Chinese Medical University, Hangzhou, China; 2 Functional Department, Haiyan People's Hospital, Jiaxing, China

**Keywords:** artificial intelligence, atrial fibrillation, catheter ablation, late recurrence, risk prediction

## Abstract

Atrial fibrillation (AF) is the most common sustained arrhythmia, and catheter ablation is an established rhythm-control strategy. However, late atrial arrhythmia recurrence remains common, limiting long-term procedural benefits. This review provides a structured and critical overview of current evidence on the predictors of late recurrence after AF catheter ablation across four domains: circulating biomarkers, imaging and electrophysiological substrate markers, genetic susceptibility, and artificial intelligence and machine-learning models. Novel predictors, including fibrosis-associated biomarkers, N-terminal pro-B-type natriuretic peptide, left atrial fibrosis quantified by late gadolinium enhancement cardiac magnetic resonance, low-voltage regions, epicardial adipose tissue features, and PITX2-associated genetic variants, may yield biological insights beyond conventional clinical scoring systems. Artificial intelligence-based approaches may additionally improve risk stratification through multimodal data integration; nevertheless, current evidence predominantly originates from retrospective single-center cohorts and is constrained by overfitting, variable rhythm-monitoring protocols, dataset shift, and inadequate external validation. Predictive performance also depends on the clinical and procedural context and may vary between paroxysmal and persistent AF, initial and repeat ablation, and thermal and pulsed-field energy modalities. Before routine implementation, future investigations should prioritize standardized phenotyping, rigorous prospective multicenter validation, transparent reporting, and clinically interpretable decision-support systems.

## 1 Introduction

Atrial fibrillation (AF) is one of the most prevalent sustained cardiac arrhythmias worldwide, with its global prevalence continuing to increase. AF adversely affects quality of life and confers major risks of ischemic stroke, heart failure, and mortality, consequently generating considerable public-health and socioeconomic burdens (1). This burden is expanding rapidly throughout developing regions, particularly Asia (2), highlighting the requirement for widely applicable and cost-effective management approaches. Among appropriately selected patients with symptomatic paroxysmal or persistent AF, catheter ablation, particularly pulmonary vein isolation (PVI), constitutes an established therapeutic approach to rhythm control (3, 4). Recent technological developments, notably pulsed-field ablation mediated by irreversible electroporation, have broadened the range of procedural approaches and may minimize collateral injury to non-cardiac structures (5).

However, recurrence of AF, atrial flutter, or atrial tachycardia beyond the post-ablation blanking period continues to represent a principal obstacle to sustained rhythm control. Late recurrence has been documented in approximately 20%–30% of patients with paroxysmal AF and 40%–50% of patients with persistent AF; however, reported rates differ depending on patient selection, ablation strategy, monitoring intensity, and the applied recurrence definition (6, 7). Recurrent arrhythmia may necessitate additional ablation, extended antiarrhythmic treatment, and greater utilization of healthcare resources. Recurrence definitions continue to evolve: although a 3-month blanking period has conventionally been applied, the 2024 international expert consensus proposed an 8-week blanking period for clinical trials and emphasized that monitoring intensity substantially affects the detection of recurrence (8).

Although traditional clinical prediction models—such as the APPLE and CAAP-AF scores—offer some utility in risk assessment, their predictive performance remains suboptimal (C-index approximately 0.65—0.75) and they fail to adequately capture individual atrial substrate heterogeneity, particularly regarding pathophysiological mechanisms such as fibrosis extent and inflammation (9). These models are primarily based on readily available clinical parameters and do not reflect the complex, dynamic nature of the AF substrate. Furthermore, recent studies have reported variability in the predictive performance of these models across racial and ethnic groups, indicating the importance of incorporating race-specific biological and socio-environmental factors into risk assessment frameworks (10).

Accordingly, rather than focusing on established clinical scoring systems, this review presents an organized critical appraisal of predictors spanning biological levels, namely circulating biomarkers, imaging-defined and electrophysiological attributes of the atrial substrate, genomic determinants, and artificial intelligence (AI) models for integrating heterogeneous datasets. The objectives are to explain the biological rationale and methodological foundations underlying these predictors, evaluate evidentiary limitations and inconsistencies, differentiate clinically meaningful ablation contexts, and examine the potential contributions of predictive tools to patient selection, procedural planning, and post-ablation surveillance.

## 2 Literature identification approach

The present article followed a structured narrative-review framework and was not conducted as a formal systematic review. Relevant publications were located by focused searches of PubMed/MEDLINE, Web of Science Core Collection, and Embase covering database inception through July 26, 2026, with search-term combinations addressing atrial fibrillation, catheter ablation, late recurrence, biomarkers, fibrosis, inflammation, imaging, electrophysiology, genetics, artificial intelligence, and machine learning. Study selection preferentially included original investigations, randomized trials, consensus statements, and authoritative reviews that contributed directly to predicting post-ablation recurrence or interpreting its clinical significance. Given that the article did not constitute a formal systematic review or meta-analysis, neither pooled estimation of effects nor structured synthesis of risk of bias was performed.

## 3 Contribution and limitations of traditional prediction models

### 3.1 Classic clinical and electroanatomical predictors

Numerous studies have established clear associations between various clinical and electroanatomical factors and the risk of AF recurrence following catheter ablation. Clinically, advanced age, male sex, persistent or long-standing persistent AF, longer AF duration, and comorbidities such as obesity (elevated body mass index), hypertension, diabetes, heart failure—particularly heart failure with preserved ejection fraction—chronic kidney disease, and obstructive sleep apnea have all been consistently associated with increased recurrence risk (11). The underlying pathophysiological mechanisms are multifactorial: aging and comorbidities contribute to atrial structural remodeling; sleep apnea induces hypoxemia and autonomic instability; and heart failure elevates atrial pressure, promoting dilation and fibrosis.

Recent studies have also identified non-alcoholic fatty liver disease and metabolic syndrome as independent predictors of AF recurrence, offering novel perspectives for risk stratification (12). These conditions are associated with systemic pro-inflammatory and profibrotic states, which may negatively affect the atrial substrate and foster AF maintenance. At the level of cardiac structure and function, increased left atrial anteroposterior diameter and elevated left atrial volume index are among the most robustly validated predictors, Additional factors such as left ventricular hypertrophy and reduced left ventricular ejection fraction are also linked to adverse outcomes. Left atrial enlargement serves as a surrogate marker for chronic atrial stretch and remodeling, while impaired LV function reflects a more advanced cardiac pathology that may extend to the atria.

In recent years, left atrial strain parameters—particularly reservoir strain—have demonstrated superior predictive value compared to traditional volume-based indices (13). As a dynamic measure of atrial function and deformation and function, left atrial strain offers deeper insights into fibrotic and stiffening processes that static volume metrics may not capture. Electroanatomical parameters directly reflect abnormalities in the atrial substrate. During electrophysiological studies, the presence and extent of left atrial low-voltage zones (commonly defined as bipolar voltage <0.5 mV) identified via high-density mapping, as well as the distribution of complex fractionated atrial electrograms, serve as direct markers of atrial fibrosis and electrical conduction heterogeneity. Their extensive presence is strongly associated with a significantly increased risk of recurrence (14). These zones reflect areas of slow conduction or conduction block that may anchor reentrant circuits or exhibit abnormal automaticity, facilitating AF initiation and persistence. Collectively, these factors form the foundation of traditional risk stratification; however, their individual predictive value is often modest and reflects a relatively static snapshot of a complex and dynamic disease process.

### 3.2 Existing clinical prediction scoring systems

To integrate diverse risk factors and provide practical tools for clinical assessment, several prediction scores have been developed. The APPLE score incorporates age, persistent AF, impaired estimated glomerular filtration rate, enlarged left atrial diameter, and reduced left ventricular ejection fraction (15), whereas alternative scoring systems integrate clinical, anatomical, and procedural parameters. Overall, these scores provide only moderate discriminatory performance, with areas under the receiver operating characteristic curve or C-indices typically between approximately 0.65 and 0.75 (16). Their principal utility lies in initial risk stratification and patient counseling rather than precise individualized prognostication. Consequently, biomarker-enhanced prediction strategies have been investigated. For example, integrating fibrosis-associated biomarkers such as Galectin-3 into clinical prediction frameworks may strengthen biological plausibility; however, their incremental predictive utility, assay standardization, and optimal cutoff values remain uncertain (17–19).

### 3.3 Limitations and unmet clinical needs

Although the aforementioned traditional prediction models and scoring systems serve as valuable tools for clinical risk assessment, their inherent limitations are increasingly evident. First, the predictive accuracy of these models remains modest, with AUC values typically ranging between 0.65 and 0.75. This level of discrimination is insufficient for reliably distinguishing between high-risk and low-risk individuals, falling short of the precision medicine standard required to accurately identify patients at elevated risk. As a result, these models may contribute to misclassification, potentially leading to suboptimal patient selection for ablation or missed opportunities to initiate more intensive adjunctive therapies in those who would benefit most.

Second, traditional models are primarily based on macroscopic clinical phenotypes and do not adequately quantify individual differences in atrial substrate which underlie the core mechanisms of AF initiation and maintenance. Key pathological features—such as the extent and distribution of myocardial fibrosis, local inflammatory activity, and autonomic nervous system remodeling—are not directly assessed. Instead, the atrium is effectively treated as a “black box,” with its internal condition inferred from external clinical markers rather than measured through direct structural or functional characterization.

Moreover, most current assessments are static, relying on pre-procedural baseline parameters. They fail to capture post-procedural dynamic changes that occur during the healing and remodeling phases, including lesion maturation, scar formation, inflammation, and reverse atrial remodeling. These time-dependent processes can significantly influence long-term outcomes, and a static, pre-ablation snapshot cannot account for the variability in individual healing responses.

In addition, traditional models entirely overlook individual genetic susceptibility, which constitutes a critical, intrinsic layer of AF risk. Genetic predisposition influences atrial architecture, electrophysiological properties, and response to injury, yet remains invisible to conventional risk models based solely on clinical and imaging data. Furthermore, most existing prediction models have been developed using data from predominantly European populations, limiting their generalizability across diverse ethnic groups. Their applicability to Asian, African, and other non-European populations requires further validation (20), as genetic background, environmental exposures, and lifestyle factors can significantly influence the relevance and weight of specific risk predictors.

Therefore, there is an urgent need for novel prediction tools that can comprehensively and accurately reflect atrial substrate characteristics and individualized risk profiles. Integrating molecular biomarkers, advanced imaging modalities, genetic data, and sophisticated computational techniques—including AI—represents a promising path toward achieving the next level of predictive precision in clinical practice.

The characteristics, predictive value, and key limitations of novel predictors across various categories—including biomarkers, imaging, electrophysiological, and genetic factors—are summarized in Table 1.

**Table 1:** Novel predictors of late AF recurrence after catheter ablation: characteristics and evidence levels.

Predictor category	Specific markers/features	Pathophysiological basis	Predictive value (Typical AUC/OR)	Key limitations
Biomarkers	Galectin-3, sST2	Myocardial fibrosis, inflammation	AUC 0.65–0.75	Lack of standardized cut-off values
	NLR, SII, hs-CRP	Systemic inflammation	OR 1.5–2.8	Non-specific, influenced by comorbidities
	NT-proBNP	Neurohormonal activation, atrial stretch	AUC 0.68–0.72	Affected by ventricular function
Imaging Features	LGE-CMR fibrosis extent	Atrial structural remodeling	AUC 0.70–0.82	Cost, availability, quantification variability
	Epicardial adipose tissue	Paracrine inflammation	OR 2.1–3.2	Measurement standardization needed
	Left atrial strain	Atrial functional remodeling	AUC 0.71–0.78	Vendor-dependent parameters
Electrophysiological	Low-voltage zones	Myocardial scar/fibrosis	OR 2.5–4.0	Mapping density/duration dependent
	Rotors/focal sources	AF drivers	OR 1.8–3.2	Mapping system differences
Genetic	PITX2 polymorphisms	Genetic susceptibility	OR 1.3–1.7	Modest individual effect size
	Polygenic risk scores	Cumulative genetic risk	AUC 0.62–0.68	Population-specific effect

sST2, Soluble Suppression of Tumorigenicity 2; NLR, Neutrophil-to-Lymphocyte Ratio; SII, systemic immune-inflammation index; Hs-CRP, High-sensitivity C-Reactive Protein; NT-proBNP, N-terminal pro-B-type natriuretic peptide; LGE-CMR, late gadolinium enhancement cardiovascular magnetic resonance; AF, atrial fibrillation; PITX2, Paried-like homeodomain transcription factor 2; AUC, area under the curve; OR, odds ratio.

## 4 Predictive value of novel biomarkers and imaging/electrophysiological features

### 4.1 Biomarkers revealing mechanisms of recurrence

Circulating biomarkers can non-invasively reflect underlying pathophysiological states and hold considerable potential for predicting late recurrence, offering insights into the mechanisms driving AF recurrence from multiple perspectives. Myocardial fibrosis biomarkers such as Galectin-3 (Gal-3) and soluble ST2 (sST2)—which mediate fibrotic processes and antagonize the anti-fibrotic effects of IL-33, respectively—have been identified as independent predictors of recurrence following AF ablation when pre-procedural serum levels are elevated (17, 18). Gal-3 is secreted by activated macrophages and promotes fibroblast proliferation and collagen deposition, while sST2 functions as a decoy receptor for IL-33, thereby inhibiting its cardioprotective and anti-fibrotic effects. Elevated levels of these markers indicate an active pro-fibrotic myocardial environment. Recent studies have shown that the combined assessment of Gal-3 and sST2 improves predictive accuracy compared to either marker alone (19). This multi-marker approach captures complementary aspects of the fibrotic pathway, offering a more comprehensive evaluation of fibrotic burden and activity.

Systemic inflammation indicators, including the Systemic Immune-Inflammation Index, Neutrophil-to-Lymphocyte Ratio, and high-sensitivity C-reactive protein, are also significantly associated with recurrence risk (21, 22). These markers reflect systemic inflammatory states that contribute to atrial electrical and structural remodeling. Inflammation promotes oxidative stress, alters ion channel function, and disrupts intercellular coupling, collectively fostering a pro-arrhythmic substrate. These hematological indices provide accessible, albeit indirect, measures of systemic inflammatory burden. Recently, novel inflammatory indicators such as the platelet-to-lymphocyte ratio and monocyte-to-HDL ratio have also demonstrated predictive potential (23). Platelet-to-lymphocyte ratio (may represent the interplay between inflammation and thrombosis, while monocyte-to-HDL ratio links innate immune activation with lipid metabolism—both pathways implicated in AF pathogenesis.

The neurohormonal biomarker N-terminal pro-brain natriuretic peptide (NT-proBNP), which reflects cardiac load and function, has consistently emerged as a strong predictor of recurrence when pre-procedural levels are elevated (24). NT-proBNP is released from cardiomyocytes in response to wall stress and serves as a sensitive marker of hemodynamic burden. Elevated levels often correlate with left atrial enlargement and dysfunction—key components of the AF substrate. Collectively, these biomarkers offer objective laboratory data to support post-ablation risk stratification, functioning as a “liquid biopsy” of underlying pathological processes that complement anatomical and functional assessments. Notably, dynamic changes in biomarkers—such as the magnitude of NT-proBNP reduction following ablation—may offer superior predictive value compared to static, single time-point measurements (25). A significant post-procedural decline may indicate effective reduction of atrial pressure or volume overload, suggesting a favorable long-term prognosis.

### 4.2 Deepening application of imaging and electrophysiological features

Advanced imaging modalities and electrophysiological methods enable comprehensive characterization of the atrial substrate. Late gadolinium enhancement cardiac magnetic resonance permits quantitative evaluation of the extent and spatial distribution of left atrial fibrosis, and findings from the DECAAF study established an independent relationship between a greater pre-ablation fibrotic burden and post-ablation arrhythmia recurrence (26). However, the prognostic significance of fibrosis should be differentiated from the therapeutic effectiveness of fibrosis-guided ablation. In the randomized DECAAF II trial, supplementing PVI with magnetic resonance-guided fibrosis ablation in patients with persistent AF did not significantly decrease atrial arrhythmia recurrence and resulted in a higher frequency of safety events (27). Accordingly, imaging-defined fibrosis should primarily be regarded as an indicator of substrate complexity and recurrence susceptibility rather than evidence that routine fibrosis-targeted ablation confers superior clinical outcomes.

Cardiac computed tomography (Cardiac CT) can precisely assess left atrial volume, pulmonary vein anatomy, and the volume and density of epicardial adipose tissue (EAT). EAT is a metabolically active tissue that secretes adipokines and pro-inflammatory cytokines capable of influencing the adjacent atrial myocardium through paracrine mechanisms. Abnormal EAT characteristics are associated with increased recurrence risk due to their pro-inflammatory and profibrotic effects. Emerging radiomics techniques enable the extraction of advanced image-based features, including texture and heterogeneity, which are imperceptible to human observers but carry predictive value (28, 29). Radiomics allows for high-throughput quantification of subtle image features that may reflect underlying tissue properties such as microfibrosis or fat infiltration. Recent research has shown that radiomic features of EAT provide superior prognostic information compared to volume measurements alone (30), suggesting that qualitative properties of EAT may better indicate biological activity and risk.

At the electrophysiological level, low-voltage zones, identified through high-density mapping, serve as direct indicators of myocardial scar or severe fibrosis. Additional features such as complex fractionated atrial electrograms and localized sources of abnormal electrical activity—including rotors and focal origins—contribute significantly to recurrence risk assessment and the development of individualized ablation strategies (31, 32). High-density mapping enables detailed electroanatomical characterization of the atrium, identifying regions of electrical silence (scar), slow conduction (border zones), and complex activation patterns that may perpetuate AF. Substrate modification strategies targeting these regions are commonly employed in persistent AF cases.

Recent advancements in high-density mapping technology have facilitated more precise and reproducible assessment of atrial electrical characteristics. Moreover, machine learning (ML)-based methods are being developed to automate classification of electrophysiological features such as complex fractionated atrial electrograms and rotational activity (33). These algorithms can analyze large volumes of electrogram data, enhancing objectivity, reducing inter-observer variability, and potentially uncovering novel electrophysiological signatures associated with recurrence risk.

## 5 Genomic and multi-omics perspectives

### 5.1 Genetic susceptibility: single nucleotide polymorphisms

AF has a strong genetic component. Genome-wide association studies have identified more than 100 genetic loci linked to AF susceptibility. Among these, single nucleotide polymorphisms in the chromosomal region 4q25, near the PITX2 (Paired Like Homeodomain 2) gene, exhibit the most significant and consistent association with both the risk of AF onset and recurrence after catheter ablation (34). PITX2 is a transcription factor essential for left–right asymmetry and pulmonary vein myocardial sleeve development during embryogenesis. Reduced PITX2 expression in adulthood is thought to create a permissive electrophysiological environment by altering ion channel expression (e.g., reducing the inward rectifier potassium current IK1), thereby increasing susceptibility to AF initiation and recurrence. The gene plays a critical role in cardiac development, particularly in left atrial myocardial differentiation and the formation of pulmonary vein sleeves. Functional impairment may destabilize atrial electrical activity and structure. In addition to PITX2, other loci—such as 16q22 (ZFHX3) and 1q21—have also been associated with recurrence risk following ablation (35). ZFHX3 is implicated in transcriptional regulation and sarcomere organization, while the 1q21 region lies near genes such as KCNN3, which encodes a potassium channel involved in atrial repolarization. These associations underscore the multiple biological pathways through which genetic factors influence AF vulnerability, including transcriptional regulation, structural maintenance, and ion channel function. Genotyping may facilitate the identification of patients with elevated intrinsic risk for recurrence, independent of conventional clinical indicators. This genetic information could inform pre-procedural decision-making, helping to identify individuals who may benefit from more extensive initial ablation or enhanced post-ablation monitoring—even in the absence of other high-risk features. Recent research has also investigated the utility of polygenic risk scores in predicting AF recurrence. High polygenic risk scores have been significantly associated with increased recurrence risk (36). A polygenic risk score aggregates the effects of multiple common single nucleotide polymorphisms across the genome into a single quantitative measure of genetic liability, offering a more comprehensive view of inherited risk than individual variants alone.

### 5.2 Towards multi-omics integration

While single-omics approaches offer valuable insights, they provide limited resolution into the complex pathophysiology of AF. A growing trend is the integration of diverse omics layers—such as genomics, transcriptomics, proteomics, and metabolomics—with clinical, imaging, and biomarker data. This multi-omics integration can construct a more comprehensive and precise risk profile for AF recurrence, uncover novel pathophysiological mechanisms, and identify potential therapeutic targets—thus representing a critical step toward true precision medicine (37).

For example, a genomic variant may confer risk only if it leads to altered gene expression (transcriptomics), which subsequently affects protein abundance (proteomics) or metabolic pathways (metabolomics). Multi-omics frameworks capture these cascades and interactions. Recent studies have combined genomic and proteomic data to identify new biological pathways associated with recurrence, including those related to extracellular matrix remodeling and immune–inflammatory processes (38). These integrative analyses can reveal downstream mediators of genetic risk—such as key proteins or signaling pathways—that are potentially modifiable by therapeutic intervention.

Moreover, the emergence of single-cell sequencing technologies enables cell-type-specific resolution of atrial remodeling processes. Traditional bulk sequencing masks heterogeneity by averaging signals across all cells in a sample. In contrast, single-cell RNA sequencing distinguishes transcriptional profiles of individual cell types—such as cardiomyocytes, fibroblasts, immune cells, and endothelial cells—within atrial tissue. This approach has provided novel insights into how specific cell populations contribute to fibrosis, inflammation, and electrical remodeling in AF (39). Such resolution is essential for the development of targeted therapies that act selectively on the cellular drivers of disease.

## 6 Integration and breakthrough of AI and ML models

### 6.1 Data integration advantages of AI models

AI-based methods, particularly ML and deep learning, enable the analysis of high-dimensional, multimodal, nonlinear datasets. Selected investigations have combined clinical characteristics, laboratory biomarkers, imaging-derived features, electrophysiological mapping measurements, and genomic information, thereby achieving stronger discrimination than conventional scoring systems (40–43). However, superior predictive accuracy is not intrinsic to AI: performance is governed by cohort size, data integrity, feature-selection procedures, endpoint specification, validation methodology, and concordance between the development sample and the target clinical population.

Graph-based neural architectures are increasingly being investigated for cardiovascular risk estimation because they can model interrelationships among patients, variables, and biological entities (44). However, evidence directly evaluating graph neural networks for post-ablation recurrence prediction remains scarce. Accordingly, their present contribution is primarily conceptual and hypothesis-generating rather than representative of established clinical applicability.

### 6.2 Representative models and predictive performance

For post-ablation recurrence prediction, random forests, support vector machines, gradient-boosting algorithms, and deep-learning models have demonstrated encouraging discrimination within selected retrospective cohorts (41–43). Although certain models have reported areas under the receiver operating characteristic curve exceeding 0.80, comparison with conventional scores remains challenging because investigations vary in AF phenotype, ablation modality, follow-up duration, rhythm-monitoring intensity, feature availability, and validation methodology. Predictive performance derived from internal cross-validation should not be regarded as evidence of reproducible superiority across external clinical populations.

Deep learning, a major subset of ML, has demonstrated particular strength in the automated analysis of medical imaging. It has been successfully applied to tasks such as automated segmentation of left atrial structures and precise quantification of fibrosis burden from CMR images. These applications enhance the objectivity and efficiency of image feature extraction, providing a solid foundation for building highly accurate predictive models (45). Convolutional neural networks, in particular, are capable of learning hierarchical imaging features—ranging from basic edges to complex textures—directly from pixel-level input. This enables the discovery of novel imaging biomarkers that may not be identifiable through conventional clinical assessments.

Recent investigations have increasingly integrated imaging-derived, electrocardiographic, clinical, and procedural variables into multimodal predictive frameworks. Explainable ML methods have additionally been applied to prioritize patient-specific determinants of recurrence risk and evaluate model behavior in independent held-out cohorts (42). Although these advances are encouraging, they remain at the proof-of-concept stage unless their predictive performance is prospectively replicated across institutions, device platforms, demographic populations, and diverse ablation workflows.

### 6.3 Clinical necessity of explainable AI

AI models—particularly deep learning models—are often criticized for their “black box” nature, as their internal decision-making processes can be difficult to interpret. This lack of transparency hinders clinician trust and limits clinical adoption. Explainable AI (XAI) addresses this limitation by enhancing interpretability. One widely used XAI technique is Shapley Additive exPlanations (SHAP), which assigns an importance value (SHAP value) to each predictive feature, clearly illustrating both the direction and magnitude of a given feature's contribution to an individual prediction (46). For instance, in a high-risk patient, SHAP analysis might reveal that an extensive low-voltage zone was the primary driver of risk, whereas a normal NT-proBNP level slightly mitigated it. This level of transparency builds clinician confidence and facilitates meaningful clinical reasoning.

Explainability may strengthen clinician confidence and facilitate biological hypothesis generation; however, feature-attribution methods neither demonstrate causality nor remain stable when predictor variables are correlated. Model calibration, decision-curve analysis, transportability, and prospective clinical impact are equally important measures of model performance. Beyond predicting recurrence, AI has been applied in cardiac electrophysiology to electrocardiographic phenotype recognition, automated electroanatomical mapping analysis, arrhythmia classification, driver identification, and procedural planning in cardiac electrophysiology (47), demonstrating a broader translational scope while underscoring the requirement for rigorous validation.

## 7 Discussion

The evidence supports a multidimensional framework for post-ablation recurrence in which clinical phenotype, atrial substrate, systemic biomarkers, inherited susceptibility, and procedural determinants interact. However, the additional predictive contribution of many emerging markers remains uncertain. Most investigations are retrospective, originate from single centers, and include relatively small cohorts; furthermore, endpoint definitions and rhythm-monitoring protocols vary, and numerous AI models depend exclusively on internal validation. Overfitting, class imbalance, approaches to missing data, selective feature availability, dataset shift, and inadequate demographic representation may exaggerate apparent model performance and impair transportability. Imaging protocols, biomarker assays, and mapping systems likewise vary among institutions, whereas financial and logistical barriers may accentuate disparities between tertiary centers and resource-constrained settings. Consequently, strong discriminatory performance in retrospective datasets alone does not warrant routine clinical implementation.

Predictive performance also varies according to clinical and procedural context. Markers of structural substrate, including extensive fibrosis, low-voltage zones, and increased left atrial dimensions, may be more informative in persistent AF than in paroxysmal AF, whereas pulmonary vein reconnection may have greater relevance in paroxysmal disease. Predictors developed in index-ablation cohorts should not be directly extrapolated to repeat procedures, for which prior lesion sets, scar patterns, and procedural indications differ. Ablation energy source represents an additional source of heterogeneity: evidence derived from radiofrequency or cryoballoon cohorts may not be directly applicable to pulsed-field ablation because lesion biology, collateral injury profiles, and healing responses differ. Future investigations should therefore report and validate predictive performance separately according to AF phenotype, index versus repeat ablation, and energy source.

Landmark clinical trials further highlight the importance of distinguishing prognostic association from therapeutic efficacy. DECAAF identified left atrial fibrosis burden as a prognostic marker, whereas DECAAF II demonstrated that routine magnetic resonance-guided fibrosis ablation added to PVI did not improve recurrence outcomes in persistent AF and was associated with more safety events (26, 27). EAST-AFNET 4 established the clinical benefits of early rhythm-control therapy in recently diagnosed AF, whereas CABANA delineated the broader advantages and limitations of catheter ablation relative to pharmacological therapy (48, 49). Although these trials inform patient selection, treatment timing, and outcome prioritization, they should not be regarded as direct validation of any particular model for predicting late recurrence.

To address current challenges and facilitate sustained progress in this field, future efforts should prioritize the following key directions.

First, large prospective multicenter cohorts with standardized definitions, imaging protocols, biospecimen handling, electroanatomical mapping, and rhythm monitoring are required. Models should be evaluated using independent external validation, calibration, decision-curve analysis, and prespecified subgroup analyses rather than discrimination alone.

Second, dynamic prediction frameworks should be investigated through the integration of longitudinal rhythm and physiological measurements obtained from implantable monitors and wearable devices. These models could continuously update recurrence risk instead of generating a single pre-ablation estimate; however, their clinical utility requires evaluation against standardized follow-up protocols and clearly defined, actionable management pathways.

Third, prediction research should progress from association toward intervention. Randomized studies are needed to determine whether management guided by a biomarker, imaging marker, genotype, or validated AI estimate improves symptoms, arrhythmia burden, repeat-procedure rates, quality of life, or other patient-centered outcomes.

Finally, decision-support systems should be interpretable, interoperable with electronic health records, and designed around clinical workflow. Reporting should address data provenance, missingness, fairness, uncertainty, calibration, and failure modes. Privacy-preserving approaches such as federated learning may support multicenter development without centralized transfer of patient-level data, although governance, harmonization, and prospective evaluation remain essential (50).

## 8 Conclusion

Late recurrence following AF catheter ablation arises from complex interactions among clinical phenotype, atrial substrate, circulating biomarkers, inherited susceptibility, and procedural context. Emerging biomarkers, advanced imaging techniques, electrophysiological mapping, and genomic profiling may provide mechanistic insights beyond conventional risk scores; however, their predictive utility varies across AF subtype, index versus repeat procedures, and ablation technologies. AI and ML broaden the potential for multimodal risk stratification, yet routine clinical implementation remains premature because external validation is limited, outcome ascertainment is heterogeneous, datasets may be biased, and model interpretability remains insufficient. Meaningful progress will require standardized definitions, prospective multicenter investigations, independent validation, and clinically actionable tools that improve decision-making and patient outcomes rather than merely demonstrate strong retrospective accuracy.
